# A novel aerial manipulator system compensation control based on ADRC and backstepping

**DOI:** 10.1038/s41598-021-01628-1

**Published:** 2021-11-16

**Authors:** Le Ma, Yiming Yan, Zhiwei Li, Jie Liu

**Affiliations:** Robotics Technology Laboratory, School of Automation and Engineering, Northeast Electric Power University, JiLin, 132012 China

**Keywords:** Computer science, Aerospace engineering

## Abstract

This paper proposes a fully-actuated control method for a novel aerial manipulation system (AMS). A customized carbon frame structure supports the servo actuators, on which eight propellers group into pairs located. We present kinematics and dynamics modeling of the AMS based on Craig parameter method and recursive Newton–Euler equation, respectively. Then, an Active disturbance rejection control (ADRC)—Backstepping—Compensation controller is designed to control the exact position and orientation of the manipulator in the Cartesian space. Finally, the performance of the system is demonstrated through simulations and virtual experiments.

## Introduction

The interest towards aerial manipulator systems (AMSs) is daily growing in many countries by each passing day. Including proper tools in an aerial platform, e.g. a gripper or robotic arms, extends the range of applications of unmanned aerial vehicles (UAVs)^1^.The vast majority of aerial operations, such as cleaning buildings, assembly of transmission lines, or the maintenance of photovoltaic equipment, still rely on labor. The risks, arduousness, and inefficiency associated with the aerial operations are apparent. Therefore, there is a need for the development of effective aerial automation equipment to upgrade existing industrial equipment and to address the demands of the industry and labor costs.

Existing research has nearly covered all of the combinations of traditional aerial platforms and different types of operating agencies. Systems that combine multiple rotors and robotic arms in series offer significant advantage over other combinations in terms of flexibility, controllability, etc.^2,3^. The motions associated with aerial platforms are relatively limited, so Euler angles are commonly used to analyze the kinematics of an aerial platform. Analysis of the motions of a robotic arm is typically conducting based on Craig parameter method. In addition, dynamic modeling of the platform and robotic arms continues to use the Newton-Euler and Lagrangian methods^4^.

Although a lot of work has been done in AMS control, little work has sufficient to support actual tasks in the industry^5^, because the complexities of the AMS. Development of control methods for aerial platforms is one of the key issues^6–8^. The difficulties are as follows:

1. The AMS belongs to a class of systems that contain tree-shaped, rootless, multiple rigid bodies, and the strong coupling and nonlinearity increase the level of difficulty when trying to control the system.
2. A dynamic constraint exists between the aerial platform and the robotic arm. The position and movement speed of the manipulator correspondingly exert substantial effects on the stability control of the aerial platform. The motion of the aerial platform in turn affects the position and attitude of the end–effector.
3. The aerial platforms that are currently used by research institutes are mostly nonintegrated structures, and the positions and postures need to be controlled by subsystems, which may sacrifice the accuracy of position or attitude control.
4. Due to the limitations of the mechanical structure, the aerial platform cannot provide sufficient compensation for the motion of the robot arm, which may affect the stability of the system. Therefore, it is necessary to develop a robust solution to the AMS control problem.

In order to improve the stability of the UAV, the mass ratio of the aerial platform to the manipulator is used to reduce the impact caused by the motion of the manipulator arm^9^, but this method also leads to a lower load capacity for the system. The system described in^10,11^ integrates a high-power redundant manipulator to increase the load capacity. However, the study focuses on trajectory planning for the manipulator and increases the complexity of the control strategy. At the same time, the equipment is too large to be suitable for conventional aerial tasks. Nguyen^12^ uses multiple platforms to carry one robotic arm to improve execution ability of the system and reduce the impact of the robotic arm on a single platform. Although this method expands the system operation space, the difficulties associated with coordination of the system also increased, which affects the overall stability of the system.

In addition to mechanical parameters, interactions between subsystems also exert an effect influence on the stability of the whole aerial platform. For this reason, Mellinger^13^ proposes a method for estimating the interaction torques generated by robotic arms, but the study only considered static conditions. With the continuous deepening studies, Jimenez-Cano and Fumagalli^14,15^ consider the AMS as an integral time-varying rigid body and design a controller using an estimate of the overall mass center and inertial tensor of the AMS during the motion of the robot arm. However, the theoretical basis and accuracy of this method with regards to the momentum theorem still remain to explore. Moreover, from the viewpoint of algorithm, it is extremely difficult to calculate the dynamic inertia tensor and the centroid of the AMS.

Yang^16^ considers the interactions between subsystems as perturbations and designed a controller accordingly. However, when the torques in the system change continuously, control of the system is limited. Kondak^17^ adopts a method of model predictive control to address the limitations of the previous study associated with perturbations, but the control performance is easily affected by the designed objective function. Furthermore, the solution methods are relatively complex and can impact real-time performance. Mebarki and Caccavale and Fanni^18–20^ establish analytical representations for the interacting torques between subsystems and use the representations to design and analyze dynamic load compensation terms. However, due to the limitations associated with the mechanical structure, existing aerial platforms cannot fully compensate for the torques that are generated by the dynamic loads in any directions.

As seem from these studies, one of the main problems in AMS control is the interaction existing in the system/subsystems. The associated control methods are the key research areas that need further research. Rajappa and Scholten^21,22^ consider the aerial platform as a complete system, then optimize the parameters of the rotor shaft, thereby improving the flexibility and controllability of the whole system. Inspired by^23^, in our previous work, we designed an AMS with an fully-actuated aerial platform based on a Dynamic Compensation–PID (DC–PID) control method^24,25^.

This paper describes a AMS with one human size arm manipulator for outdoor operation. Whereas most AMS that can be found in literatures are prototypes evaluated in the lab, the proposed AMS structure overcomes the issues of underactuation of the standard multirotor drones for aerial manipulation. The main contributions of this paper are as follows:

1. We design a novel AMS for aerial manipulation (eight propellers group into pairs that are rotating about different axes) which allows to independently reach positions and orientations in 3D dimension.
2. With the continuous deepening of our research, the performance of the end–effector and the interactions between the manipulator and aerial platform is improved by combining ADRC and Backstepping control techniques (referred as ABC control).

The manipulator is built with eight smart servo actuators and a customized carbon frame that reduces the self weight without sacrificing stiffness of AMS. Four typical static positions in the operation of the robotic arm are evaluated in simulation analysis. The paper also addresses its integration in an aerial platform, including position and attitude control. The kinematics and dynamics of the AMS is derived, proposing a control scheme that makes use of the manipulator dynamics for compensating the reaction wrenches. The interactions between the manipulator and the aerial platform are experimentally identified in test bench in hovering conditions.

## Mechanical structure

**Figure 1:**
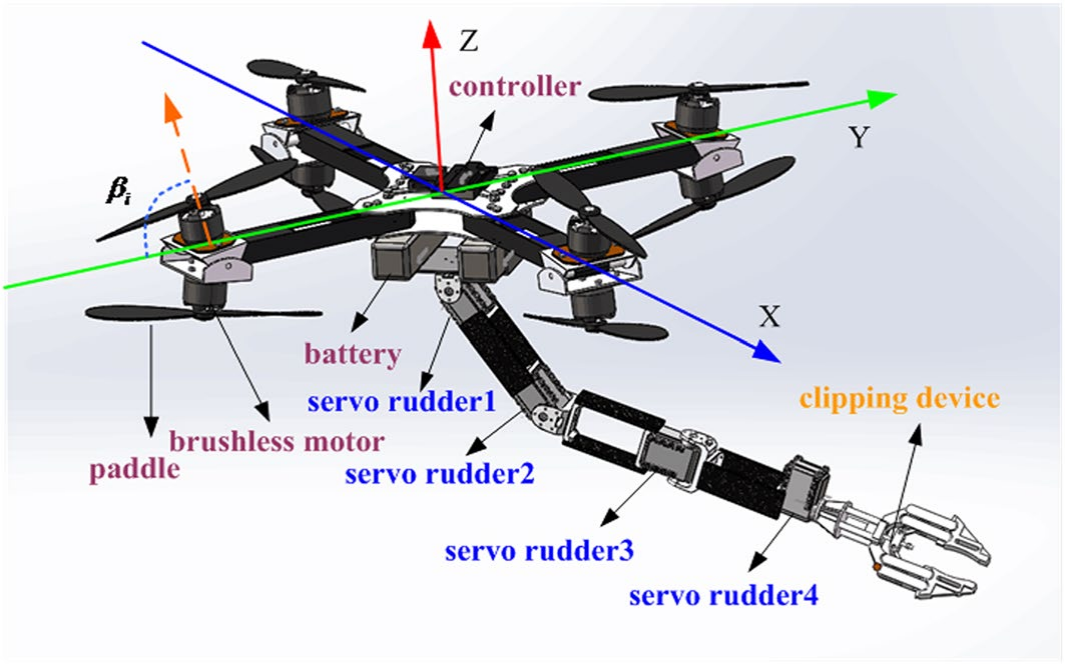
Structure of the fully-actuated AMS.

Figure 1 shows the design of the fully-actuated AMS in SolidWorks. The main difference between the present design and the design of traditional aerial platforms is that the rotor structure proposed in this paper possess a large angle of inclination (${\beta _i}<60^ \circ$). Therefore, the rotor can provide propulsion torques and moments in the *X* and *Y* directions of the body coordinate system because of which the fully-actuated drive for the aerial platform can be realized.

**Figure 2:**
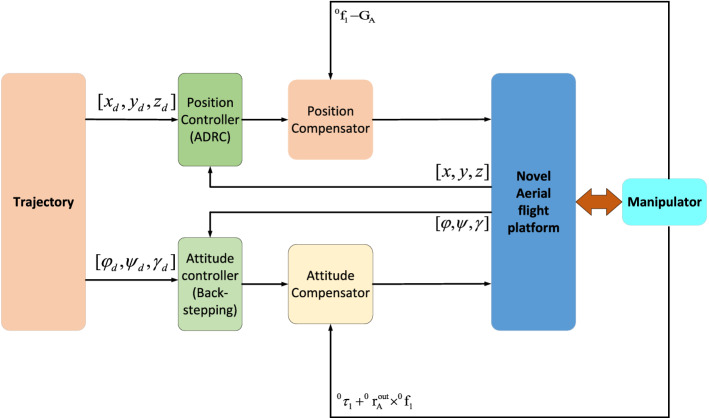
Control structure of the fully-actuated AMS.

In this system, the robotic arm is designed to have 4 joints to balance flexibility and load capacity. At the joints, Dynamixel AX-12A servo rudders are used as joint drives. The servos provide feedback including position, speed, and other information. Therefore, by combining the feedback with voltage, current, load and other feedback information, the angular acceleration of the joint can be estimated. The position and speed can then be synthesized into instructions, and the motion of the joint can be controlled.The control structure of the Novel Aerial Manipulator System is shown in Fig. 2. It is composed of compensators and a ADRC controllers and Back-stepping controllers. ADRC controllers are designed for position system. Backstepping controllers are designed for attitude system. Compensators are added into position system and attitude system to compensate the influence of the force of the manipulator on the system.

The inertial reference coordinate system, the aerial platform coordinate system, and the manipulator’s *i*th joint coordinate system are ${\mathrm{\{ W\} }}$, ${\mathrm{\{ A\} }}$, and ${\mathrm{\{ M_i\} }}$, respectively. The pose matrix of ${\mathrm{\{ A\} }}$ versus ${\mathrm{\{ W\} }}$ is

1 $$\begin{aligned} ^{\mathrm{0}}{{{\mathbf {T}}}_{{\mathbf {A}}}} = \left[ {\begin{array}{*{20}{c}} {^0{{{\mathbf {R}}}_{\mathrm{A}}}}&{}{^0{{{\mathbf {P}}}_{\mathrm{A}}}}\\ {{{{\mathbf {0}}}_{1 \times {\mathrm{3}}}}}&{}1 \end{array}} \right] \end{aligned}$$

where ${^0{{{\mathbf {P}}}_{\mathrm{A}}}}$ and ${^0{{{\mathbf {R}}}_{\mathrm{A}}}}$ are the rotation matrices represented by the origin of ${\mathrm{\{ A\} }}$ in the ${\mathrm{\{ W\} }}$ vector, and ${^0{{{\mathbf {R}}}_{\mathrm{A}}}}$ can be obtained from the Euler angle $\left[ {\varphi ,\psi ,\gamma } \right]$ in the X–Y–Z coordinate system.

The transformation matrix between the $i-1$ and *i* joint coordinate systems of the manipulator can be expressed as the following formula (specifying $i=1$ as the transformation matrix of joint systems 1 and ${\mathrm{\{ A\} }}$).

2 $$\begin{aligned} ^{i - 1}{{{\mathbf {M}}}_i} = \left[ {\begin{array}{*{20}{c}} {^{i - 1}{{{\mathbf {R}}}_i}}&{}{^{i - 1}{{{\mathbf {P}}}_i}}\\ {{{{\mathbf {0}}}_{1 \times {\mathrm{3}}}}}&{}1 \end{array}} \right] \end{aligned}$$

where $^{i - 1}{{{\mathbf {R}}}_i}$ is the rotation matrix that the *i* coordinate system transforms into the $i-1$ coordinate system, and $^{i - 1}{{{\mathbf {P}}}_i}$ is the representation of the origin in the *i* coordinate system in the $i-1$ coordinate system. We can use Craig’s parameter $\left\langle {{\alpha ,\mathbf {a,d},\theta }} \right\rangle$ to calculate $^{i - 1}{{{\mathbf {M}}}_i}$, where $\left\langle {{\alpha ,\mathbf {a,d}}} \right\rangle$ is a constant and ${{\theta }}$ is a variable that represents the angle of the joint.

As shown in Table 1, the Craig configuration parameters for the robotic arm described in this paper (in order to conveniently represent the position and position of the end–effector, using the concept of a virtual joint, where ${\theta _5}\equiv 0$).

**Table 1:** Craig parameters for aerial manipulator.

${{{\varvec{\alpha }}}} /^\circ$	${{\mathbf {a}}}/^\circ$	${{\mathbf {d}}}/^\circ$	${{{\varvec{\theta }}}}/^\circ$
0	0	0	${\theta _1}$
0	${l_1}$	0	${\theta _2}$
$-\pi {\mathrm{/2}}$	${l_1}$	0	${\theta _3}$
$-\pi {\mathrm{/2}}$	${l_3}$	0	${\theta _4}$
0	0	${l_4}$	${\theta _5}\equiv 0$

From the transformation between coordinate systems, the position and attitude matrix of each joint coordinate system relative to the $\left\{ {\mathrm{W}} \right\}$ coordinate system can be obtained as:

3 $$\begin{aligned} ^{\mathrm{0}}{{{\mathbf {T}}}_i} = \left[ {\begin{array}{*{20}{c}} {^0{{{\mathbf {R}}}_i}}&{}{{}^0{{{\mathbf {P}}}_i}}\\ 0&{}1 \end{array}} \right] = {}^0{{{\mathbf {T}}}_{\mathrm{A}}}\prod \limits _k^i {^{k- 1}{{{\mathbf {M}}}_k}} \end{aligned}$$

The relationship between the origin of the joint coordinate system and the center of mass of the connecting rod in the $\left\{ {\mathrm{W}} \right\}$ coordinate system and the vectors $^0{{{\mathbf {P}}}_i}$ and $^0{{\mathbf {P}}}_i^{\mathrm{c}}$ can be expressed as:

4 $$\begin{aligned} \left\{ \begin{array}{l} ^0{{{\mathbf {P}}}_i} = {}^0{{{\mathbf {P}}}_{i - 1}} + {}^0{{{\mathbf {R}}}_{i - 1}}{}^{i - 1}{{{\mathbf {P}}}_i}\\ ^0{{\mathbf {P}}}_i^{\mathrm{c}} = {}^0{{{\mathbf {P}}}_i} + {}^0{{{\mathbf {R}}}_i}{}^i{{\mathbf {P}}}_i^{\mathrm{c}} \end{array} \right. \end{aligned}$$

where $^i{{\mathbf {P}}}_i^{\mathrm{c}}$ is the centroid of the link *i* in *i* coordinate system. The relationship between the angular velocity and its derivative on the aerial platform is as follows:

5 $$\begin{aligned} \left[ \begin{array}{l} {{{\dot{\phi }}} }\\ {{{\dot{\psi }}} }\\ {{{\dot{\gamma }}} } \end{array} \right] = \left[ {\begin{array}{*{20}{c}} 1&{}{\sin \phi \tan \psi }&{}{ \cos \phi \tan \psi }\\ 0&{}{\cos \phi }&{}{ - \sin \phi }\\ 0&{}{\frac{{\sin \phi }}{{\cos \psi }}}&{}{\frac{{\cos \phi }}{{\cos \psi }}} \end{array}} \right] {}^0{{\varvec{\omega }}_{\mathrm{A}}} \end{aligned}$$

The angular velocity of each joint coordinate system is determined by $^0{{\varvec{\omega }}_{\mathrm{A}}}$ and the joint angular velocity of the joint ${{{{\dot{\theta }}} }}$. The recurrence relationship between the parameters m is as follows:

6 $$\begin{aligned} ^0{{\varvec{\omega }}_i} = {}^0{{\varvec{\omega }}_{i{\mathrm{- }}1}} + {}^0{{{\mathbf {R}}}_i}{{{{{\dot{\theta }}} }}_i}{{{\mathbf {Z}}}_0} \end{aligned}$$

where, $${{{\mathbf {Z}}}_0} = [ {0,0,1} ]^{\mathrm{T}}$$.

Derivation of (4) can be used to describe the relationships between the linear velocities in each joint coordinate system.

7 $$\begin{aligned} \left\{ \begin{aligned} ^0{{{\dot{\mathbf {P}}}}_i}&= {}^0{{{\dot{\mathbf {P}}}}_{i{\mathrm{- 1}}}} + {}^0{\varvec{\omega }_{i{\mathrm{- 1}}}} \times {}^0{{{\mathbf {R}}}_{i - 1}}{}^{i{\mathrm{- 1}}}{{{\mathbf {P}}}_i}\\ ^0{\dot{\mathbf {P}}}_i^{\mathrm{c}}&= {}^0{{{\dot{\mathbf {P}}}}_i} + {}^0{{\varvec{\omega }}_i} \times {}^0{{{\mathbf {R}}}_i}{}^i{{\mathbf {P}}}_i^{\mathrm{c}} \end{aligned} \right. \end{aligned}$$

Equations (1)–(7) establish the relationships among the position, attitude, and speed between each link of the manipulator and the aerial platform in the coordinate system.

In this paper, a recursive Newton-Euler equation is used to establish the dynamic model of the system and the variables are all expressed in the $\left\{ {\mathrm{W}} \right\}$ coordinate system. The power equation for the aerial platform is as follows:

8 $$\begin{aligned} \left\{ \begin{aligned} {}^0{{{\mathbf {I}}}_{\mathrm{A}}}{}^0{{{\varvec{{{\dot{\omega }}} }}}_{\mathrm{A}}} + {}^0{{\varvec{\omega }}_{\mathrm{A}}} \times {}^0{{{\mathbf {I}}}_{\mathrm{A}}}{}^0{{\varvec{\omega }}_{\mathrm{A}}}&= {}^0{{{\mathbf {U}}}_{{\tau }}} - {}^0{{{\tau }}_1} - {}^0{{\mathbf {r}}}_{\mathrm{A}}^{{\mathrm{out}}} \times {}^0{{{\mathbf {f}}}_1}\\ {m_{\mathrm{A}}}{}^0{{{{{\ddot{\mathbf {P}}}}}}_{\mathrm{A}}}&= {}^0{{{\mathbf {U}}}_{\mathrm{f}}} - {}^0{{{\mathbf {f}}}_{\mathrm{1}}} + {{{\mathbf {G}}}_{\mathrm{A}}} \end{aligned} \right. \end{aligned}$$

$^0{{{\mathbf {U}}}_{\mathrm{f}}}$ and ${}^0{{{\mathbf {U}}}_{{\tau }}}$ are respectively the driving force and torque generated by the rotor. $^0{{{\mathbf {f}}}_1}$ and ${}^0{{{\tau }}_1}$ are respectively the force and torque of the aerial platform acting on the link 1. ${}^0{{\mathbf {r}}}_{\mathrm{A}}^{{\mathrm{out}}}$ is a vector representation of the arm that acts on link 1. ${m_{\mathrm{A}}}$ is the mass of the aerial platform, and ${}^0{{{\mathbf {I}}}_{\mathrm{A}}}$ is the inertial tensor of the aerial platform relative to $\left\{ {\mathrm{W}} \right\}$. ${{{\mathbf {G}}}_{\mathrm{A}}}$ is the vector representation for gravity acting on the aerial platform.

One of the dynamic characteristics of an AMS is that the platform interacts with the robot arm. Thus, calculation of $^0{{{{\ddot{\mathbf {P}}}}}_{{\mathbf {A}}}}$ and ${}^0{\varvec{{{\dot{\omega }}}}_{\mathrm{A}}}$ requires ${}^0{{{\mathbf {f}}}_1}$ and ${}^0{{{\tau }}_1}$. However, ${}^0{{{\mathbf {f}}}_1}$ and ${}^0{{{\tau }}_1}$ are also determined by the former. Therefore, by considering the dynamics of the entire link, (9) is the Newton-Euler equation for link *i*.

9 $$\begin{aligned} \left\{ \begin{aligned} {}^0{{{\mathbf {I}}}_i}{}^0{{{\varvec{{\dot{\omega }}}}}_i}&= {}^0{{{\tau }}_i} + {}^0{{\mathbf {r}}}_i^{{\mathrm{in}}} \times {}^0{{{\mathbf {f}}}_i}\\&\quad - {}^0{{{\tau }}_{i + 1}} - {}^0{{\mathbf {r}}}_i^{{\mathrm{out}}} \times {}^0{{{\mathbf {f}}}_{i + 1}}\\&\quad - {}^0{{\varvec{\omega }}_i} \times {}^0{{{\mathbf {I}}}_i}{}^0{{\mathbf {\omega }}_i}\\ m_i^{\mathrm{m}}{}^0{{{\ddot{\mathbf {P}}}}}_i^{\mathrm{c}}&= {}^0{{{\mathbf {f}}}_i} - {}^0{{{\mathbf {f}}}_{i + 1}} + {{\mathbf {G}}}_i^{\mathrm{m}} \end{aligned} \right. \end{aligned}$$

${}^0{{{\mathbf {f}}}_i}$ and ${}^0{{{\tau }}_i}$ are the force and torque of link $i - 1$ relative to link *i*. ${m_i}$ is the mass of the link, and ${}^0{{{\mathbf {I}}}_i}$ is the inertial tensor of the link relative to $\left\{ {\mathrm{W}} \right\}$. ${{\mathbf {G}}}_i^{\mathrm{m}}$ represents the gravity vector associated with link *i*. ${}^0{{\mathbf {r}}}_i^{{\mathrm{in}}}$ and ${}^0{{\mathbf {r}}}_i^{{\mathrm{out}}}$ are respectively the force arm vectors of link $i - 1$ and link *i* relative to link *i* and link $i+1$ .

10 $$\begin{aligned} \left\{ \begin{aligned} {}^0{{\mathbf {r}}}_i^{{\mathrm{in}}}&= {}^0{{{\mathbf {P}}}_i} - {}^0{{\mathbf {P}}}_i^{\mathrm{c}}\\ {}^0{{\mathbf {r}}}_i^{{\mathrm{out}}}&= {}^0{{{\mathbf {P}}}_{i + 1}} - {}^0{{\mathbf {P}}}_i^{\mathrm{c}} \end{aligned} \right. \end{aligned}$$

The time derivative of (6) and (7) can be obtained as follows:

11 $$\begin{aligned} \left\{ \begin{aligned} ^0{{{\varvec{{{\dot{\omega }}} }}}_i}&= {}^0{{{\varvec{{{\dot{\omega }}} }}}_{i{\mathrm{- 1}}}} + {}^0{{\varvec{\omega }}_i} \times {}^0{{{\mathbf {R}}}_i}{{{{{{\dot{\theta }}} }}}_i}{{\mathbf {\mathrm{Z}}}_0} + {}^0{{{\mathbf {R}}}_i} {{{{\ddot{\theta }}}}_i}{{{\mathrm{Z}}}_0}\\ ^0{{{\ddot{{\mathbf {P}}}}}_i}&= {}^0{{{\ddot{{\mathbf {P}}}}}_{i{\mathrm{- 1}}}} + {}^0{{{\varvec{{{\dot{\omega }}} }}}_{i{\mathrm{- 1}}}} \times {}^0{{{\mathbf {R}}}_{i - 1}}{}^{i{\mathrm{- 1}}}{{{\mathbf {P}}}_i}\\&\quad + {}^0{{\varvec{\omega }}_{i{\mathrm{- 1}}}} \times \left( {{}^0{{\varvec{\omega }}_{i{\mathrm{- 1}}}} \times {}^0{{{\mathbf {R}}}_{i - 1}}{}^{i{\mathrm{- 1}}}{{{\mathbf {P}}}_i}} \right) \\ ^0{\ddot{{\mathbf {P}}}}_i^{\mathrm{c}}&= {}^0{{{\ddot{{\mathbf {P}}}}}_i} + {}^0{{{\varvec{{{\dot{\omega }}} }}}_i} \times {}^0{{{\mathbf {R}}}_i}{}^i{{\mathbf {P}}}_i^{\mathrm{c}}\\&\quad + {}^0{{\varvec{\omega }}_i} \times \left( {{}^0{{\varvec{\omega }}_i} \times {}^0{{{\mathbf {R}}}_i}{}^i{{\mathbf {P}}}_i^{\mathrm{c}}} \right) \end{aligned} \right. \end{aligned}$$

The simultaneous expressions of (8)–(11) can be used to form second-order algebraic differential equations. The equation set can be solved by taking the second-order term as a variable and the other parameters as constants.

## Methods

From the analysis above, we can see that the dynamic model of the aerial platform is a MIMO, second-order nonlinear model, and it has strong coupling with aerial manipulator system. In comparison to traditional multi-rotor platforms, the stability of the aerial platform in an AMS is greatly affected by the motion of the robotic arm. Therefore, this paper uses the characteristics of the dynamic model to establish a dynamic compensation term to offset this effect. The position and attitude controllers of the aerial platform are designed based on the ADRC and Backstepping control respectively.

### Compensation for the dynamic load

Based on (8), it can be concluded that the acceleration and angular acceleration of the platform centroid are related to the angular velocity and manipulator force/torque in addition to the driving force/torque control. In this paper, the force/torque of the manipulator is regarded as the load on the platform and the compensation term ${{{\mathbf {u}}}_{\mathrm {dc}}}$ is established to reduce its side effects on the system. ${{{\mathbf {u}}}_{{\mathrm {dc}}}}$ is defined as:

12 $$\begin{aligned} {{{\mathbf {u}}}_{{\mathrm{dc}}}} = \left[ \begin{array}{l} {}^0{{{\mathbf {f}}}_{\mathrm{1}}} - {{{\mathbf {G}}}_{{\mathbf {A}}}}\\ {}^0{{{\tau }}_1} + {}^0{{\mathbf {r}}}_{\mathrm{A}}^{{\mathrm{out}}} \times {}^0{{{\mathbf {f}}}_1} \end{array} \right] \end{aligned}$$

By simultaneously solving (8)–(11), the required compensation parameters can be obtained. Details can be found in the our previous works^24^. Since the control input is introduced with this compensation term, the model of the platform dynamics can be simplified. Therefore, the platform control problem for the AMS is transformed into a traditional multirotor control problem. The input form of the controller is as follows:

13 $$\begin{aligned} {{\mathbf {u}}} = {{{\mathbf {u}}}_{{\mathrm {ap}}}} + {{{\mathbf {u}}}_{{\mathrm {dc}}}} \end{aligned}$$

where ${{{\mathbf {u}}}_{\mathrm {ap}}}$ is the controller input for the rotor platform. The disturbance to the platform position has an effect on the attitude stability. In this paper, the position and attitude controllers for the platform are designed using ADRC and Backstepping control.

### ADRC for determination of platform position

The position ${{{\mathbf {P}}}_{\mathrm{A}}}$ and velocity ${{{\dot{\mathbf {P}}}}_{\mathrm{A}}}$ of the aerial platform are assumed to be the system states $\left[ {{{\mathbf {x}}}_1^{\mathrm{p}}{\mathbf {,x}}_2^{\mathrm{p}}} \right]$. After the compensation term is eliminated, the position term in (8) can be simplified as follows:

14 $$\begin{aligned} \left\{ \begin{array}{l} {{\dot{\mathbf {x}}}}_1^{\mathrm{p}} = {{\mathbf {x}}}_2^{\mathrm{p}}\\ {{\dot{\mathbf {x}}}}_2^{\mathrm{p}} = \frac{1}{{{m_{\mathrm{A}}}}}{{{\mathbf {u}}}_{\mathrm{a}}} \end{array} \right. \end{aligned}$$

Equation (14) describes a MIMO second-order system. The following section describes the ADRC controller designed in this paper. The ADRC controller consists of a tacking differentiator (TD), which is based on the transition process, an extended state observer (ESO), and a nonlinear feedback control law.

The discrete form of the second-order nonlinear fastest TD can be expressed as follows:

15 $$\begin{aligned} \left\{ \begin{array}{l} {{\mathbf {v}}}_1^{k + 1} = {{\mathbf {v}}}_1^k + h{{\mathbf {v}}}_2^k\\ {{\mathbf {v}}}_2^{k + 1} = {{\mathbf {v}}}_2^k + h{f_{{\mathrm{han}}}} ({{\mathbf {v}}}_1^k - {{\mathbf {y}}}_{\mathrm{d}}^k,{{\mathbf {v}}}_2^k|r,{h_0}) \end{array} \right. \end{aligned}$$

where *h* is the sampling time, ${{{\mathbf {y}}}_{\mathrm{d}}}$ is the control expectation, ${{{\mathbf {v}}}_1}$ and ${{{\mathbf {v}}}_2}$ are the input of the transition process and the differential signal tracked by the input, respectively, and ${f_{{\mathrm{han}}}}$ is the special function:

16 $$ f_{{han}} (x_{1} ,x_{2} |r,h_{0} ) = \left\{ {\begin{array}{*{20}l}    { - r\text{sgn} (a),\left| a \right|} \hfill & {>\,d} \hfill  \\    {r\frac{a}{d},\left| a \right| \le d} \hfill & {} \hfill  \\   \end{array} } \right.;\left\{ {\begin{array}{*{20}l}    {d = rh_{0} ,d_{0}  = h_{0} d} \hfill  \\    {y = x_{1}  + h_{0} x_{2} } \hfill  \\    {a = \left\{ {\begin{array}{*{20}l}    {x_{2}  + \frac{{a_{0}  - d}}{2},} & {\left| y \right|>\,d_{0} } \\    {x_{2}  + \frac{y}{{h_{0} }}}, & {\left| y \right| \le d_{0} }  {} \hfill  \\   \end{array} } \right.} \hfill  \\    {a_{0}  = \sqrt {d^{2}  + 8r\left| y \right|} } \hfill  \\   \end{array} } \right.$$

The ESO estimates the state of the system according to the observation output of the system, and its second-order discrete form is as follows:

17 $$\begin{aligned}&\left\{ \begin{array}{l} {{{\mathbf {e}}}^k} = {{\mathbf {z}}}_1^k - {{\mathbf {y}}}_{\mathrm{h}}^k\\ {{\mathbf {z}}}_1^k = {{\mathbf {z}}}_1^k + h({{\mathbf {z}}}_2^k - {\beta _{01}}{{{\mathbf {e}}}^k})\\ {{\mathbf {z}}}_2^k = {{\mathbf {z}}}_2^k + h({{\mathbf {z}}}_3^k - {\beta _{02}}{f_{{\mathrm{al}}}}({{{\mathbf {e}}}^k},{\alpha _1},\delta ){{{\mathbf {e}}}^k})\\ {{\mathbf {z}}}_3^k = {{\mathbf {z}}}_3^k - h{\beta _{03}}{f_{{\mathrm{al}}}}({{{\mathbf {e}}}^k},{\alpha _2},\delta ) \end{array} \right. \end{aligned}$$

18 $$\begin{aligned}&{f_{{\mathrm{al}}}}(e,\alpha ,\delta ) = \left\{ \begin{array}{l} {\left| e \right| ^\alpha }{{\mathrm{sgn}}} (e),\left| e \right| > \delta \\ \frac{e}{{{\delta ^{1 - \alpha }}}},\left| e \right| \le \delta \end{array} \right. \end{aligned}$$

where $\beta$, $\alpha$, $\delta$ are all adjustable parameters.

The nonlinear state error feedback is used as the control output for the ADRC in this paper and has the form as follows:

19 $$\begin{aligned} {{{\mathbf {u}}}_{\mathrm{a}}}= & {} \frac{{{{{\mathbf {u}}}_0} - {{{\mathbf {z}}}_{N + 1}}}}{{{b_0}}} \end{aligned}$$

20 $$\begin{aligned} {{{\mathbf {u}}}_0}= & {} \sum \limits _{i = 1}^N {{k_i}{f_{{\mathrm{al}}}}({{{\mathbf {e}}}_i},\alpha _i^{\mathrm{u}},{\delta ^{\mathrm{u}}})} \end{aligned}$$

where ${b_0}$, $\alpha _i^{\mathrm{u}}$ and ${\delta ^{\mathrm{u}}}$ are adjustable parameters. In addition, $n=2$ in a second-order system.

### Backstepping control for platform attitude

A second-order MIMO system can be represented as:

21 $$\begin{aligned} \left\{ \begin{array}{l} {\dot{\mathbf {x}}}_1^{\mathrm{b}} = {{\mathbf {f}}}_1^{\mathrm{b}}({{\mathbf {x}}}_1^{\mathrm{b}}) + {{\mathbf {g}}}_1^{\mathrm{b}}({{\mathbf {x}}}_1^{\mathrm{b}}){{\mathbf {x}}}_2^{\mathrm{b}}\\ {\dot{\mathbf {x}}}_2^{\mathrm{b}} = {{\mathbf {f}}}_2^{\mathrm{b}}({{\mathbf {x}}}_1^{\mathrm{b}},{{\mathbf {x}}}_2^{\mathrm{b}}) + {{\mathbf {g}}}_2^{\mathrm{b}}({{\mathbf {x}}}_1^{\mathrm{b}},{{\mathbf {x}}}_2^{\mathrm{b}}){{{\mathbf {u}}}_{\mathrm{b}}} \end{array} \right. \end{aligned}$$

In order to design its Backstepping controller, firstly define the error in the system as:

22 $$\begin{aligned} \left\{ \begin{array}{l} {{\mathbf {z}}}_1^{\mathrm{b}} = {{\mathbf {x}}}_1^{\mathrm{d}} - {{\mathbf {x}}}_1^{\mathrm{b}}\\ {{\mathbf {z}}}_2^{\mathrm{b}} = {{\mathbf {v}}}_1^{\mathrm{b}}({{\mathbf {x}}}_1^{\mathrm{b}}) - {{\mathbf {x}}}_2^{\mathrm{b}} \end{array} \right. \end{aligned}$$

where ${{\mathbf {x}}}_1^{\mathrm{d}}$ is the expected value of ${{\mathbf {x}}}_1^{\mathrm{b}}$, and ${{\mathbf {v}}}_1^{\mathrm{b}}$ is its virtual control input. A first-order system as follows:

23 $$\begin{aligned} {\dot{\mathbf {x}}}_1^{\mathrm{b}} = {{\mathbf {f}}}_1^{\mathrm{b}}({{\mathbf {x}}}_1^{\mathrm{b}}) + {{\mathbf {g}}}_1^{\mathrm{b}}({{\mathbf {x}}}_1^{\mathrm{b}}){{\mathbf {v}}}_1^{\mathrm{b}} \end{aligned}$$

We have selected the Lyapunov function as follows:

24 $$\begin{aligned} {V_1}({{\mathbf {z}}}_1^{\mathrm{b}}) = \frac{1}{2}{\left\| {{{\mathbf {z}}}_1^{\mathrm{b}}} \right\| ^2} \end{aligned}$$

The time derivative (22) and (24) are substituted into (23) to yield:

25 $$\begin{aligned} \left. \begin{aligned} {{\dot{V}}_1}&= {({{\mathbf {z}}}_1^{\mathrm{b}})^{\mathrm{T}}}{\dot{\mathbf {z}}}_1^{\mathrm{b}}\\&= {({{\mathbf {z}}}_1^{\mathrm{b}})^{\mathrm{T}}}\left( {{\dot{\mathbf {x}}}_1^{\mathrm{d}} - {\dot{\mathbf {x}}}_1^{\mathrm{b}}} \right) \\&= {({{\mathbf {z}}}_1^{\mathrm{b}})^{\mathrm{T}}}\left( {{\dot{\mathbf {x}}}_1^{\mathrm{d}} - {{\mathbf {f}}}_1^{\mathrm{b}}({{\mathbf {x}}}_1^{\mathrm{b}}) + {{\mathbf {g}}}_1^{\mathrm{b}}({{\mathbf {x}}}_1^{\mathrm{b}}){{\mathbf {v}}}_1^{\mathrm{b}}} \right) \end{aligned} \right. \end{aligned}$$

P-type control is adopted, so ${{\mathbf {v}}}_1^{\mathrm{b}}$ is taken as follows:

26 $$\begin{aligned} {{\mathbf {v}}}_1^{\mathrm{b}} = {({{\mathbf {g}}}_1^{\mathrm{b}})^{ - 1}}\left( {{{\dot{\mathbf {x}}}}_1^{\mathrm{d}} - {{\mathbf {f}}}_1^{\mathrm{b}} + {{\alpha }}_1^{\mathrm{b}} \circ {{\mathbf {z}}}_1^{\mathrm{b}}} \right) \end{aligned}$$

where $^i{{\alpha }}_1^{\mathrm{b}} > 0$, and $\circ$ denotes the Hadamard product. Thus, ${\dot{V}_1}({{\mathbf {z}}}_1^{\mathrm{b}} |{{\alpha }}_1^{\mathrm{b}}) = - {{\alpha }}_1^{\mathrm{b}} \circ {({{\mathbf {z}}}_1^{{\mathrm{b}}} )^{\mathrm{T}}}{{\mathbf {z}}}_1^{{\mathrm{b}}} < 0$, which means that ${{\mathbf {v}}}_1^{\mathrm{b}}$ is stable for the first-order system. Taking the system represented by (21) into account, we have selected the Lyapunov function as follows:

27 $$\begin{aligned} {V_2}({{\mathbf {z}}}_1^{\mathrm{b}},{{\mathbf {z}}}_2^{\mathrm{b}}) = \frac{1}{2}\left( {{{\left\| {{{\mathbf {z}}}_1^{\mathrm{b}}} \right\| }^2} + {{\left\| {{{\mathbf {z}}}_2^{\mathrm{b}}} \right\| }^2}} \right) \end{aligned}$$

According to the (22), the time derivative of ${{\mathbf {z}}}_1^{\mathrm{b}}$ is as follows:

28 $$\begin{aligned} \left. \begin{aligned} {\dot{\mathbf {z}}}_1^{\mathrm{b}}{\mathrm{= }}{{{\dot{\mathbf {x}}}}^{\mathrm{d}}} - {\dot{\mathbf {x}}}_1^{\mathrm{b}}&= {{{\dot{\mathbf {x}}}}^{\mathrm{d}}} - {{\mathbf {f}}}_1^{\mathrm{b}} - {{\mathbf {g}}}_1^{\mathrm{b}}{{\mathbf {x}}}_2^{{\mathbf {b}}}\\&\Rightarrow \\ {{\mathbf {x}}}_2^{{\mathbf {b}}}&= {({{\mathbf {g}}}_1^{\mathrm{b}})^{ - 1}}\left( {{{{\dot{\mathbf {x}}}}^{\mathrm{d}}} - {{\mathbf {f}}}_1^{\mathrm{b}} - {\dot{\mathbf {z}}}_1^{\mathrm{b}}} \right) \end{aligned} \right. \end{aligned}$$

Substitute (26) and (28) in (22) gives the following expression:

29 $$\begin{aligned} \left. \begin{aligned} {{\mathbf {z}}}_2^{\mathrm{b}}&= {({{\mathbf {g}}}_1^{\mathrm{b}})^{ - 1}}\left( {{\dot{\mathbf {x}}}_1^{\mathrm{d}} - {{\mathbf {f}}}_1^{\mathrm{b}} + {\mathbf {\alpha }}_1^{\mathrm{b}} \circ {{\mathbf {z}}}_1^{\mathrm{b}}} \right) \\&\quad - {({{\mathbf {g}}}_1^{\mathrm{b}})^{ - 1}}\left( {{{{\dot{\mathbf {x}}}}^{\mathrm{d}}} - {{\mathbf {f}}}_1^{\mathrm{b}} - {\dot{\mathbf {z}}}_1^{\mathrm{b}}} \right) \\&\Rightarrow \\ {{\mathbf {g}}}_1^{\mathrm{b}}{{\mathbf {z}}}_2^{\mathrm{b}}&= {\mathbf {\alpha }}_1^{\mathrm{b}} \circ {{\mathbf {z}}}_1^{\mathrm{b}}{\mathrm{+ }}{\dot{\mathbf {z}}}_1^{\mathrm{b}} \end{aligned} \right. \end{aligned}$$

The time derivative of a in (22) is then substituted into (21) to obtain the following expression:

30 $$\begin{aligned} \left. \begin{aligned} {\dot{\mathbf {z}}}_2^{\mathrm{b}}&= {\dot{\mathbf {v}}}_1^{\mathrm{b}} - {\dot{\mathbf {x}}}_2^{\mathrm{b}}\\&={\dot{\mathbf {v}}}_1^{\mathrm{b}} - {{\mathbf {f}}}_2^{\mathrm{b}} - {{\mathbf {g}}}_2^{\mathrm{b}}{{{\mathbf {u}}}_{\mathrm{b}}} \end{aligned} \right. \end{aligned}$$

The substitution eliminates ${{\mathbf {x}}}_1^{\mathrm{b}}$, ${{\mathbf {x}}}_2^{\mathrm{b}}$ and the associated derivative terms. The time derivative of ${V_2}$ is then calculated and substituted into (29) and (30) to get (31):

31 $$\begin{aligned} \left. \begin{aligned} {{\dot{V}}_2}&= {({{\mathbf {z}}}_1^{\mathrm{b}})^{\mathrm{T}}}{{\dot{\mathbf {z}}}}_1^{\mathrm{b}}{\mathrm{+ }}{({{\mathbf {z}}}_2^{\mathrm{b}})^{\mathrm{T}}}{{\dot{\mathbf {z}}}}_2^{\mathrm{b}}\\&={({{\mathbf {z}}}_1^{\mathrm{b}})^{\mathrm{T}}}\left( {{{\mathbf {g}}}_1^{\mathrm{b}}{{\mathbf {z}}}_2^{\mathrm{b}} - {\mathbf {\alpha }}_1^{\mathrm{b}} \circ {{\mathbf {z}}}_1^{\mathrm{b}}} \right) \\&\quad + {({{\mathbf {z}}}_2^{\mathrm{b}})^{\mathrm{T}}}\left( {{\dot{\mathbf {v}}}_1^{\mathrm{b}} - {{\mathbf {f}}}_2^{\mathrm{b}} - {{\mathbf {g}}}_2^{\mathrm{b}}{{{\mathbf {u}}}_{\mathrm{b}}}} \right) \\&= - {{\alpha }}_1^{\mathrm{b}}{\left\| {{{\mathbf {z}}}_1^{\mathrm{b}}} \right\| ^2}{\mathrm{+ }}{({{\mathbf {z}}}_2^{\mathrm{b}})^{\mathrm{T}}}{({{\mathbf {g}}}_1^{\mathrm{b}})^{\mathrm{T}}}{{\mathbf {z}}}_1^{\mathrm{b}}\\&\quad +{({{\mathbf {z}}}_2^{\mathrm{b}})^{\mathrm{T}}}\left( {{\dot{\mathbf {v}}}_1^{\mathrm{b}} - {{\mathbf {f}}}_2^{\mathrm{b}} - {{\mathbf {g}}}_2^{\mathrm{b}}{{{\mathbf {u}}}_{\mathrm{b}}}} \right) \end{aligned} \right. \end{aligned}$$

while

32 $$\begin{aligned} {{{\mathbf {u}}}_{\mathrm{b}}} = {({{\mathbf {g}}}_2^{\mathrm{b}})^{ - 1}}\left( {{{\dot{\mathbf {v}}}}_1^{\mathrm{b}} - {{\mathbf {f}}}_2^{\mathrm{b}} + {{\alpha }}_2^{\mathrm{b}} \circ {{\mathbf {z}}}_2^{\mathrm{b}} + {{({{\mathbf {g}}}_1^{\mathrm{b}})}^{\mathrm{T}}}{{\mathbf {z}}}_1^{\mathrm{b}}} \right) \end{aligned}$$

Then, Eq. (31) can also be expressed as follows:

33 $$\begin{aligned} {\dot{V}_2} = - {{\alpha }}_1^{\mathrm{b}}{\left\| {{{\mathbf {z}}}_1^{\mathrm{b}}} \right\| ^2} - {{\alpha }}_2^{\mathrm{b}}{\left\| {{{\mathbf {z}}}_2^{\mathrm{b}}} \right\| ^2} < 0 \end{aligned}$$

Therefore, it can be concluded that ${{{\mathbf {u}}}_{\mathrm{b}}}$ in (32) is stable relative to the system in (21).

The Euler angle vector ${{{\mathbf {e}}}_{{\mathrm{euler}}}} = {\left[ {\varphi ,\psi ,\gamma } \right] ^{\mathrm{T}}}$ and the angular velocity $^0{{\varvec{\omega }}_{\mathrm{A}}}$ of the platform are represented as the system states $\left[ {{{\mathbf {x}}}_1^{\mathrm{b}},{{\mathbf {x}}}_2^{\mathrm{b}}} \right]$ , and the following equation set is set according to (8):

34 $$\begin{aligned} \left\{ \begin{aligned} {{\mathbf {f}}}_1^{\mathrm{b}}&={{{\mathbf {0}}}_{3 \times 1}}\\ {{\mathbf {g}}}_1^{\mathrm{b}}&={{\mathbf {J}}}({{\mathbf {x}}}_1^{\mathrm{b}})\\ {{\mathbf {f}}}_2^{\mathrm{b}}&={}^0{{\mathbf {I}}}_{\mathrm{A}}^{ - 1}\left( {{{\mathbf {x}}}_2^{\mathrm{b}} \times {}^0{{{\mathbf {I}}}_{\mathrm{A}}}{{\mathbf {x}}}_2^{\mathrm{b}}} \right) \\ {{\mathbf {g}}}_2^{\mathrm{b}}&={{{\mathbf {I}}}_{3 \times 3}} \end{aligned} \right. \end{aligned}$$

where ${\mathbf {J}}$ is given by (5), and ${{{\mathbf {I}}}_{3 \times 3}}$ is a 3 dimensional unit matrix; thus, the portion of (8) that corresponds to the posture can be converted to (21). Therefore, the aerial attitude control law for the aerial platform can be obtained by (32).

## Simulation analysis

### Setup

To improve the efficiency of the simulation and to avoid copyright issues, the software used for the simulation system in this paper was changed from MATLAB to an open-source project based on Python. The main modules are shown in Table 2. (Note: Since the project is in a transitional period, the simulation program and data processing part of the study still utilize MATLAB).

**Table 2:** Main module of the simulation system.

No.	Project	Function
1	Anaconda	Development environment of Python
2	PyQt	Designing the interface of simulation program
3	Panda3D	Engine of 3D
4	Matplotlib	Displaying data
5	Numpy	Processing data and calculation
6	Blender	Importing 3D models

### Control test for a fully-actuated drive

The angles $\beta _i$, with $i (i=1, 2,\ldots , n)$ being the number of *i*–th propeller attached to the frame, can be adjusted during the preflight setup. This gives the possibility to realize a fully-actuated drive. The objective of this section is therefore to test method described in “Mechanical structure section”.

**Figure 3:**
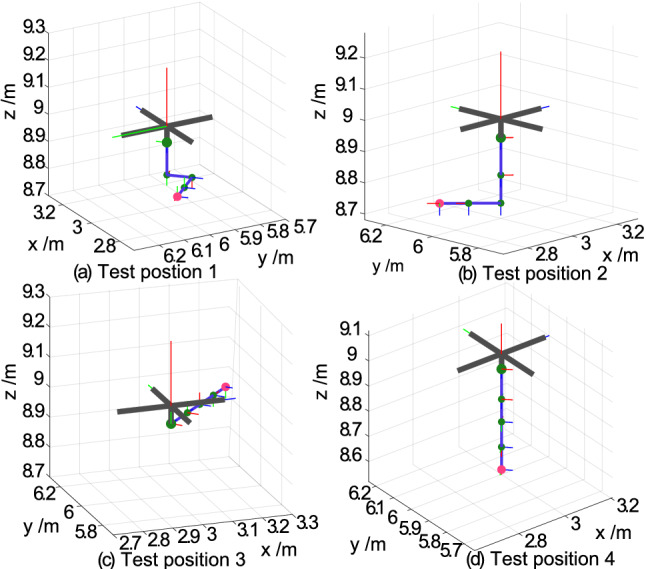
Illustration of 4 test positions of the manipulator.

**Figure 4:**
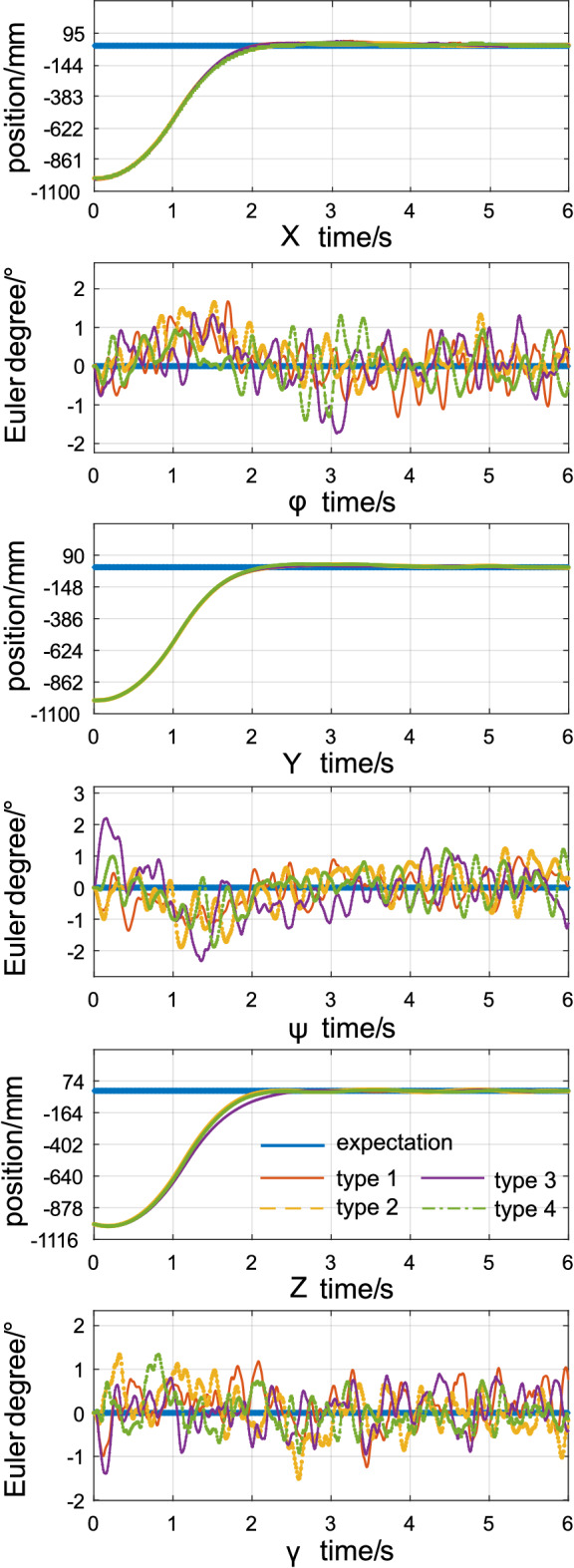
Errors associated with the control of 4 types of arm configurations.

Four representative robotic arm static positions are shown in Fig. 3. The positions are $[ - {90^ \circ }, - {90^ \circ },{0^ \circ },{0^ \circ }]$, $[ - {90^ \circ }, {0^ \circ },{0^ \circ },{0^ \circ }]$, $[ {0^ \circ }, {0^ \circ },-{90^ \circ },{0^ \circ }]$, and $[ -{90^ \circ }, {0^ \circ },-{90^ \circ },{0^ \circ }]$. First, the AMS is tested for fully-actuated drive control for position. The initial position is [9 m, 9 m, 9 m]. The expected position is [10 m, 10 m, 10 m]. The initial value and expected Euler angle are $[{0^ \circ },{0^ \circ },{0^ \circ }]$. The uncertainty associated with the parameters that are used to simulate the reference model is $\pm 10\%$. Compared with the previous work, the amplitude of the simulated noise increased with a Gaussian distribution within $\pm 30$ mm and $\pm 3^\circ$ and had a Gaussian disturbance distribution from $\pm 10$ N$\cdot$S to $\pm 80$ N$\cdot$S (referred as disturbance condition *D*0). The interval time between the disturbances is 1 s. The frequency for the simulation control is set to 100 Hz. To evaluate the performance of the ABC control on proposed AMS prototype, we perform the hovering experiment. The results are shown in Fig. 4. During the experiment, the aerial platform moves from initial position to expectation position, the position tracking error without overshoot. The aerial platform can converge to expectation position in the *X*, *Y* and *Z* directions within 2 seconds without any overshoot, and the curve associated with the displacement error is relatively smooth. The reason is that the TD term (19, 20) in the ADRC acts to remove noise.

During the control process, the error generated by the three attitude angles for the platform is always within $\pm 2^\circ$, which is less than the noise associated with the simulated sensor. Therefore, the Backstepping attitude controller designed in this paper can maintain attitude stability with a minor error in displacement control. To summarize, under different static manipulator positions and noise and disturbance conditions, the proposed method can achieve 3 dimensional position control without sacrificing attitude and therefore the fully–actuated drive position control.

**Figure 5:**
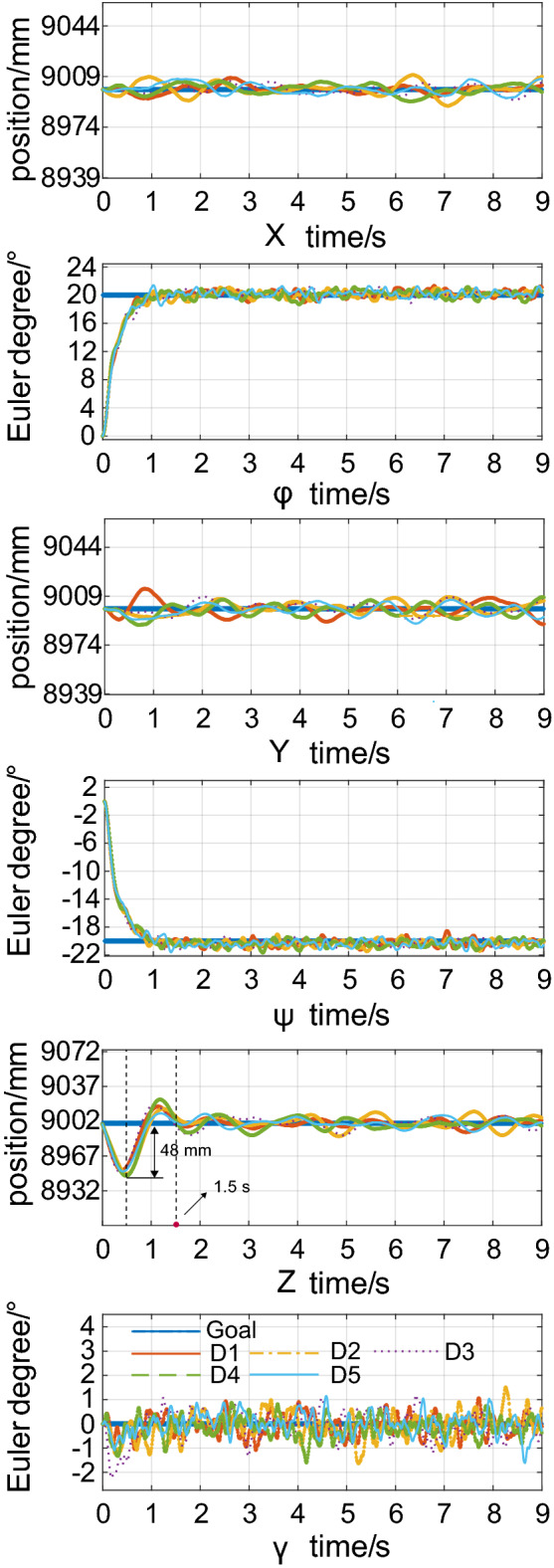
Response curves for tilt hovering control at different disturbance levels ($D1=D0, D2=2D0, D3=3D0, D4=4D0, D5=5D0$).

An additional simulation is conducted with the arm position set to $[ - {90^ \circ }, - {90^ \circ },{0^ \circ },{0^ \circ }]$. When hovering control is performed with a tilt angle, we set the disturbance condition D0 is used as the benchmark, other five types of disturbance from D1 to D5 are applied to test the performance of the ABC control method, respectively. Both the initial position and the desired position are [9 m, 9 m, 9 m], the initial Euler angle is $[{0^ \circ },{0^ \circ },{0^ \circ }]$, and the Euler angle is $[{20^ \circ },{-20^ \circ },{0^ \circ }]$. The rest of the simulation parameters are the same as above. Figure 5 shows the response curves for tilt hovering control for different levels of disturbance. The attitude angles, both $\varphi$ and $\psi$, can converge quickly (adjustment time<1 s) to expected values. At different disturbance levels, the Euler angle can maintain a steady state with only a minor error ($\pm 1.5^\circ$). Both the *X* and *Y* directions can be stabilized at a desired position with minor deviations. Due to the step response, random disturbance shock, parameter limitation of the rotor power of the system and gravity constraints, there was downward turbulence in the *Z* direction at the initial time, and the maximum oscillating amplitude is 48 mm. However, the controller can still achieve stable regulation within 1.5 seconds and maintain low error stability. In summary, the method proposed in this paper possess a faster response time and smaller error associated with the fully-actuated drive control and tilting hovering. Furthermore, the control strategy offers better noise and disturbance suppression.

### Simulation of an aerial operation

Actual aerial operations will likely require robotic arm movements. Therefore, this paper simulates two types of aerial work tasks and compares them with the methods described in previous studies^24^. The performance of this method for aerial missions involving manipulator motion was analyzed. Compared with the setting described in^24^, this paper improves the disturbance range from $\pm 40$ N$\cdot$S to $\pm 300$ N$\cdot$S.

Task 1 is the hovering control with the arm motion. The initial joint angle for the robotic arm is $[ - {90^ \circ }, {0^ \circ },-{45^ \circ },{0^ \circ }]$, the initial and expected Euler angles are both $0^ \circ$, and joint 3 moves at a speed of ${{{\dot{\theta }}} _3}{\mathrm{= }}0.5\sin t$. The range of noise and model parameter uncertainty are the same as above.

**Figure 6:**
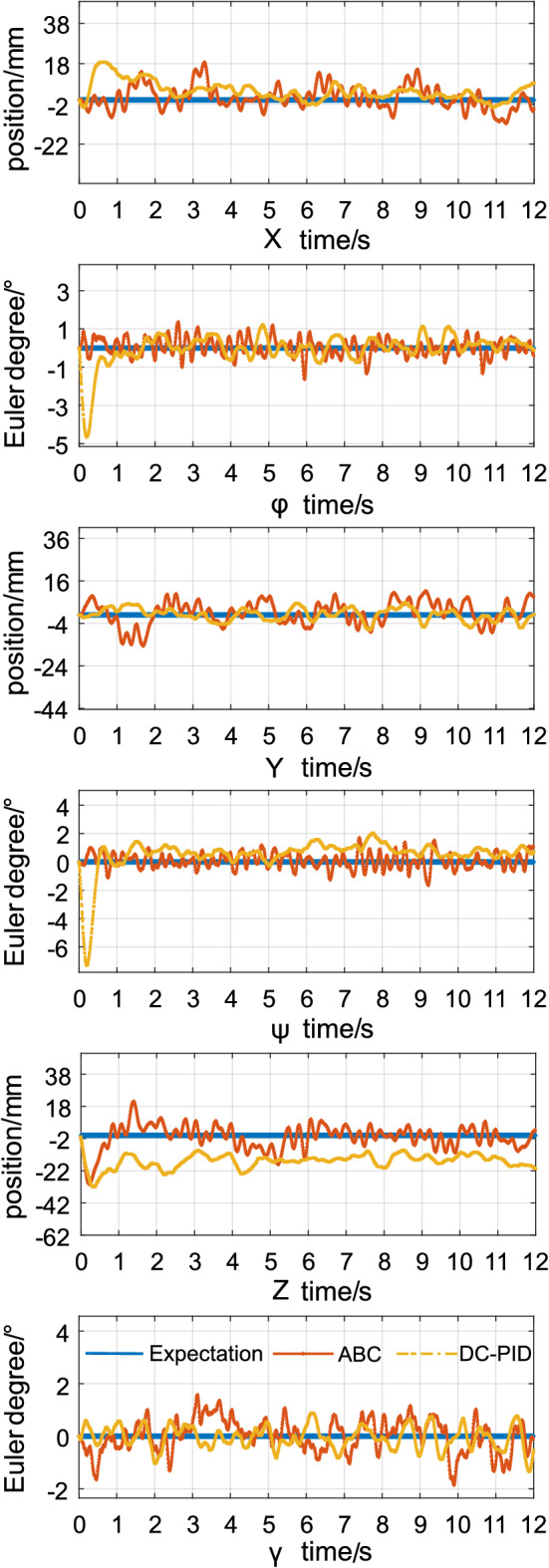
Errors in positions and attitudes for task 1.

Figure 6 shows the position and attitude error curves generated by the method (ABC) introduced in this study and the method (DC-PID) described in^24^ under the same conditions. In the *X* and *Y* directions, the control effects from the two methods are essentially the same, and all systems remain stable with small errors (within $\pm 15$ mm). Due to power limitations, initial disturbances and gravity constraints, both methods generate oscillations in the direction opposite of the *Z*-axis. The method proposed in^24^ also showed some degrees of steady–state error (approximately 20 mm), but the method described in this study can still complete an adjustment within one second and reach a stable state with a small error. The conclusions are consistent with the simulation results from the previous section. Both methods can be stable in the $\pm 1.5^\circ$ error range for a given $\gamma$ angle. Compared with the method proposed in this paper, the method described in^24^ has obvious oscillations at the $\varphi$ and $\psi$ angles at the initial time because of the movement of the robot arm and other large disturbance factors.The mean errors of the attitude angle and position are quantized as shown in Table 3. In summary, both methods can achieve stable control when performing task 1, but the method proposed in this paper has better disturbance immunity and dynamic performance in the presence of large disturbances.

**Table 3:** The comparison for control statistical indicators of task 1.

	x(m)	y(m)	z(m)	${{\phi (\circ )}}$	${{\psi (\circ )}}$	${{\gamma (\circ )}}$
Mean tracking error(ABC)	8.1293	6.9763	9.2476	0.7839	0.8127	1.3721
Mean tracking error(SPNN-PID)	8.3764	6.5372	17.4371	1.1237	1.3927	1.3687

Task 2 aims to make the aerial platform perform fully–actuated drive movement under the above conditions. The initial position and the desired position are set to [9 m, 9 m, 9 m] and [10 m, 10 m, 10 m], respectively, and the remaining parameters are the same as in task 1.

**Figure 7:**
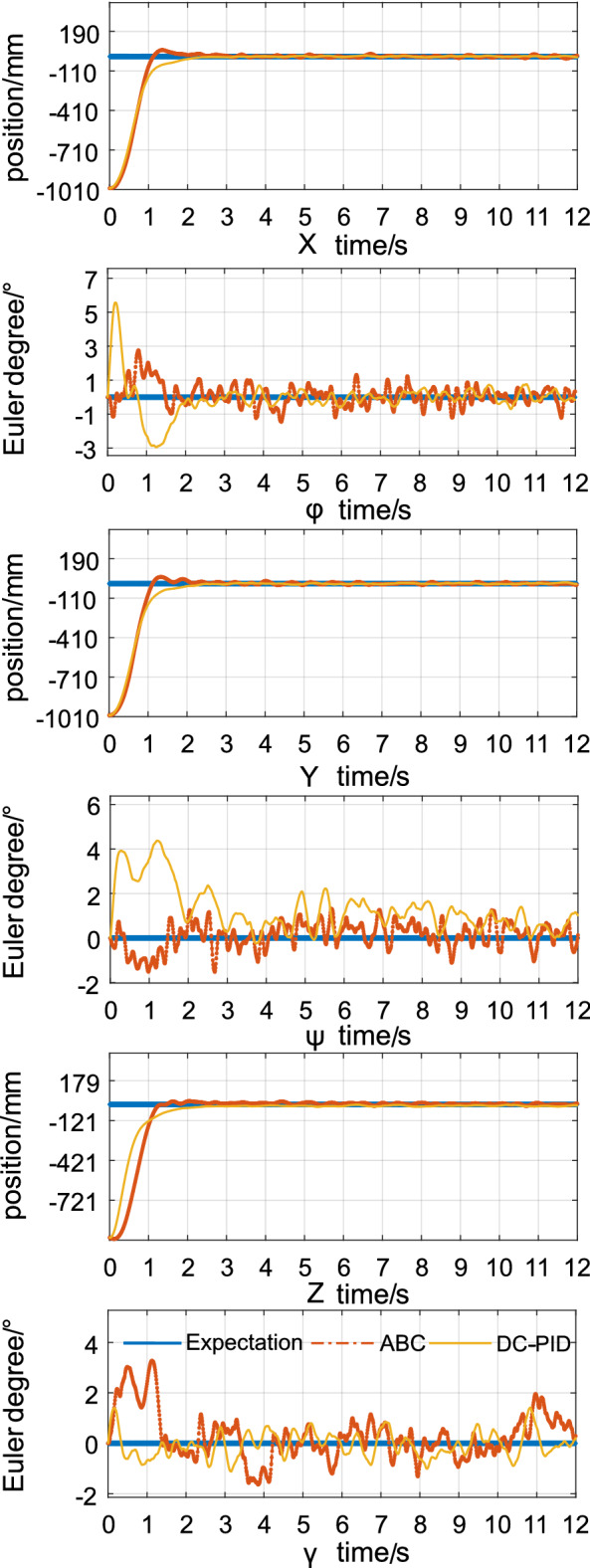
Errors in positions and attitudes for task 2.

Figure 7 shows the position and attitude error curves for the two methods. The method provided in this paper possess a faster adjustment time in the *X* and *Y* directions than the method described in^24^. Moreover, the key advantage of this method is more pronounced in terms of the response speed in the Z direction. Similar to task 1, the method described in this paper is significantly less turbulent in the initial stage of adjustment for the and corners than the method provided in^24^. Since the conditions are more complicated than in task 1 and the system performance is limited, there is likely more turbulence in the initial stage of angle adjustment than in the previous task. However, this method can achieve correction in a shorter time, and its steady-state error is equivalent to the method described in^24^. The mean errors of the attitude angle and position are quantized as shown in Table 4.

**Table 4:** The comparison for control statistical indicators of task 2.

	x(m)	y(m)	z(m)	${{\phi (\circ )}}$	${{\psi (\circ )}}$	${{\gamma (\circ )}}$
Mean tracking error(ABC)	61.1927	62.8347	63.7347	0.8743	1.4741	1.8796
Mean tracking error(SPNN-PID)	61.2453	62.8463	61.6792	1.2694	1.9763	1.5437

In summary, the method proposed in this paper is better at performing task 2 than the method provided in^24^. Based on the results from the simulations, it can be concluded that the method proposed in this paper has better disturbance suppression ability and stability than the method described in our previous study. Thus, this method has better control performance under larger noise and disturbance conditions.

## Virtual experiments

**Figure 8:**
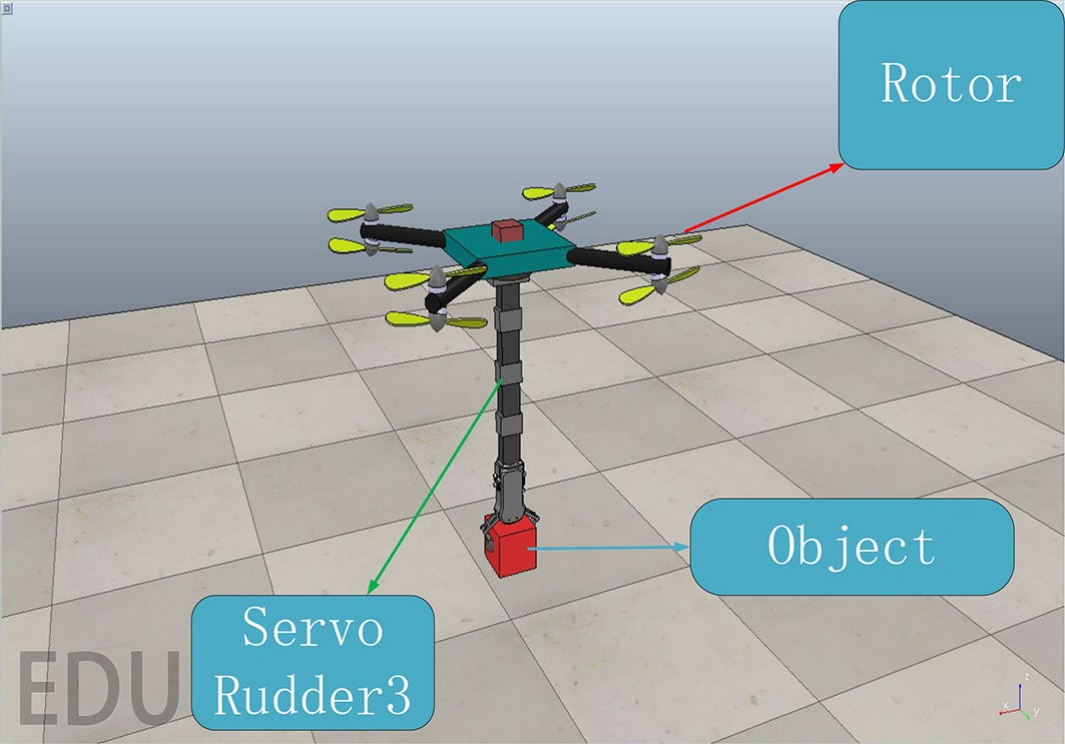
The demonstration of virtual experiment in CoppeliaSim.

The virtual experiment platform was built with coppeliasim physical simulation system. As shown in the Fig. 8. In this paper, the simulation control cycle is 0.01s. The physics engine selects Bullet 2.78. Set the precision to “highest precision”. On the basis of the previous numerical simulation, this section focuses on verifying the effectiveness of this method in practical problems. The initial position is [5 m, 5 m, 5 m]. The expected position is [6 m, 6 m, 6 m]. The initial value and expected Euler angle are$[{0^ \circ },{0^ \circ },{0^ \circ }]$. the tertiary joint swings periodically from $-\frac{\pi }{4}$ to $\frac{\pi }{4}$ while the tertiary joint moves at a speed of ${{{\dot{\theta }}} _3}{\mathrm{= }}0.5\sin t$. The range of noise and model parameter uncertainty are the same as above.

**Figure 9:**
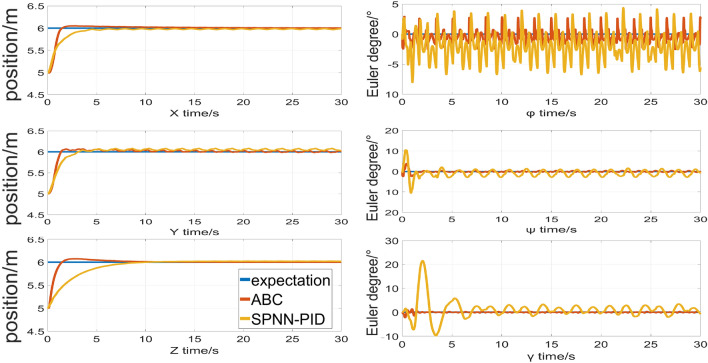
Responses in positions and attitudes for Virtual Experiments.

Figure 9 shows the position and attitude responses curves generated by the ABC control method introduced in this study and the method (SPNN-PID) described in^27^ under the same conditions. As can be seen from Fig. 9, both of the two methods can converge to the desired signal in the directions of x, y, z. However, the ABC method converges to the desired signal in about 3 seconds. On the other hand, it takes 4-6 seconds for SPNN-PID to converge to the desired signal. From this, it can be seen that the ABC method has a faster response speed.In both methods, the angle of $\varphi$ appeared a certain degree of concussion because of the robotic arm load which is tested in this section. Due to the continuous movement of joint 3, the force will be continuously transferred to the flight platform. Under the action of this continuous strong disturbance, the oscillation range of ABC method is even smaller.The average and maximum errors of the attitude angle and position are quantized as shown in Table 5.To sum up, the two methods can achieve stability control, but ABC method has better anti-disturbance ability and dynamic performance under the condition of strong disturbance.

**Table 5:** The comparison for control statistical indicators of virtual experiments.

	x(m)	y(m)	z(m)	${{\phi (\circ )}}$	${{\psi (\circ )}}$	${{\gamma (\circ )}}$
Mean tracking error(ABC)	0.0424	0.0464	0.0318	0.8267	0.2922	0.1203
Mean tracking error(SPNN-PID)	0.0529	0.0834	0.0903	2.5841	1.4954	2.4064
Maximum tracking error(ABC)	1.0	1.0	1.0	2.9306	3.9241	2.2242
Maximum tracking error(SPNN-PID)	1.0	1.0	1.0	8.0317	10.6025	21.4412

## Conclusions

In this paper, we presented a novel AMS prototype, which consists of four pairs rotors connected to a customized frame under a large angle of inclination (${\beta _i} < {60^ \circ }$, with $i=1, 2,\ldots , 8$). Three relevant conclusions are derived from the analysis of the simulation and virtual experiments.

1. This proposed AMS prototype including a 4-DOF robotic arm can overcome the well-known issues of underactuation of AMS for aerial manipulation, allowing for more robust operation, dynamic control for smoother interaction between subsystems, as compared to the systems based on the conventional AMSs. system proposed in our previous study.
2. In this paper, we provide theoretical framework for this AMS prototype, particularly for its modeling and control. Compared with DC–PID method proposed in our previous study, the ABC control method exhibit better control performance in the conditions of large noise and disturbance conditions.
3. The virtual experiments demonstrate that the method proposed in this paper can achieve stable motion, position, and attitude control under the influence of the interactions between the arm and the aerial platform.

As future work, authors are working in the optimizing the mechanical structure of the AMS prototype. This gives the possibility to adopt various manipulators in performing desired manipulation tasks in the industry. In addition, we are also proceeding in the direction of flexible adjusting parameters based on learning control method during the pre-flight setup, which is a major objective for the robust control for the AMS.

## References

[1] Ruggiero F, Lippiello V, Ollero A (2018). Aerial manipulation a literature review. IEEE Robot. Autom. Lett..

[2] Kim, S., Seo, H. & Kim, H. J. Operating an unknown drawer using an aerial manipulator. In *ICRA*, Seattle, USA, 5503–5508 (2015).

[3] Liu GS, Jia JQ (2012). UAV applications and development in the power system. J. Northeast Dianli Univ..

[4] Kim S, Seo H, Kim HJ (1959). Infrared navigation-part I: An assessment of feasibility. IEEE Trans. Electron Dev..

[5] Navarro AS, Grosch P, Lippiello V (2017). Uncalibrated visual servo for unmanned aerial manipulation. IEEE ASME T. Mech..

[6] Orsag M, Korpela CM, Bogdan S (2013). Hybrid adaptive control for aerial manipulation. J. Intell. Robot. Syst..

[7] Kobilarov M (2013). Nonlinear trajectory control of multi-body aerial manipulators. J. Intell. Robot. Syst..

[8] Orsag M, Korpela C, Bogdan S (2017). Dexterous aerial robots-mobile manipulation using unmanned aerial systems. IEEE Trans. Robot..

[9] Pounds P, Dollar AM (2014). Stability of helicopters in compliant contact under PD-PID control. IEEE Trans. Robot..

[10] Uber, F., Kondak, K., Krieger, K. et al., First Analysis and experiments in aerial manipulation using fully actuated redundant robot arm. In *IROS*, Tokyo, Japan, 3452–3457 (2013).

[11] Kondak, K., Huber, F., Schwarzbach, M. et al., Aerial manipulation robot composed of an autonomous helicopter and a 7 degrees of freedom industrial manipulator. In *ICRA*, Hong Kong, China, 2107–2112 (2014).

[12] Nguyen H, Park S, Park J (2018). A novel robotic platform for aerial manipulation using quadrotors as rotating thrust generators. IEEE T. Robot..

[13] Mellinger, D., Lindsey, Q., Shomin, M. et al., Design, modeling, estimation and control for aerial grasping and manipulation. In *IROS*, San Francisco, USA, 2668–2673 (2011).

[14] Jimenez-Cano, A. E., Martin, J. G., Heredia, G. et al., Control of an aerial robot with multi-link arm for assembly tasks. In *ICRA*, Karlsruhe, Germany, 4916–4921 (2013).

[15] Fumagalli M, Naldi R, Macchelli A (2014). Developing an aerial manipulator prototype: Physical interaction with the environment. IEEE Robot. Autom. Mag..

[16] Yang, H. & Lee, D. Dynamics and control of quadrotor with robotic manipulator. In *ICRA*, Hong Kong, China, 5544–5549 (2014).

[17] Kondak, K. *et al.* Closed-loop behavior of an autonomous helicopter equipped with a robotic arm for aerial manipulation tasks. *Int. J. Adv. Robot. Syst.* **10**(145), (2013).

[18] Mebarki R, Lippiello V (2014). Image-based control for aerial manipulation. Asian J. Control.

[19] Caccavale, F., Giglio, G., Muscio, G. et al., Adaptive control for UAVs equipped with a robotic arm. In *IFAC*, Cape Town, South Africa, 11049–11054 (2014).

[20] Fanni M, Khalifa A (2017). A new 6-DOF quadrotor manipulation system design, kinematics, dynamics and control. IEEE ASME T. Mech..

[21] Rajappa, S., Ryl, M., Bulthoff, H. H. et al., Modeling, control and design optimization for a fully-actuated hexarotor aerial vehicle with tilted propellers. In *ICRA*, Seattle, USA, 4006–4013 (2015).

[22] Scholten, J. L. J., Fumagalli, M., Stramigioli, S. et al., Interaction control of an UAV endowed with a manipulator. In *ICRA*, Piscataway, USA, 4910–4915 (2013)

[23] Nikou, A., Gavridis, G. C. & Kyriakopoulos, K. J. Mechanical design, modelling and control of a novel aerial manipulator. In *ICRA*, Seattle, USA, 4698–4703 (2015).

[24] Le M, Dong W, Yunxin L (2018). Design, modeling and dynamic compensation control of fully-actuated aerial manipulator. Chin. J. Sci. Instrum..

[25] Le, M., Yanxun, C., Jiapeng, L. et al., A hierarchical learning control framework for an aerial manipulation system, presented at the $$4^{th}$$ ICMMR, Xi’an, China, Jun. 20–24 (2017).

[26] Salazar S, Romero H, Lozano R (2009). Modeling and real-time stabilization of an aircraft having eight rotors. J. Intell. Robot. Syst..

[27] Jiang F, Pourpanah F, Hao Q (2019). Design, implementation and evaluation of a neural network based quadcopter UAV system. IEEE Trans. Ind. Electron.

